# Transplant Trial Watch

**DOI:** 10.3389/ti.2022.10365

**Published:** 2022-03-28

**Authors:** John O’Callaghan

**Affiliations:** ^1^ University Hospitals Coventry and Warwickshire, Coventry, United Kingdom; ^2^ Centre for Evidence in Transplantation, University of Oxford, Oxford, United Kingdom

**Keywords:** kidney transplantation, Vaccination, COVID-19 vaccine, randomised controlled trial, Belatacept conversion

**Randomised Controlled Trial 1**

**Randomized Trial of a Third Dose of mRNA-1273 Vaccine in Transplant Recipients**

*by Hall, V. G., et al. New England Journal of Medicine 2021; 385 (13): 1244–1246.*

**Randomised Controlled Trial 2**

**Conversion from Calcineurin Inhibitor to Belatacept-based Maintenance Immunosuppression in Renal Transplant Recipients: a Randomized Phase 3b Trial**

*by Budde, K., et al. Journal of the American Society of Nephrology 2021 [record in progress].*

To keep the transplantation community informed about recently published level 1 evidence in organ transplantation ESOT and the Centre for Evidence in Transplantation have developed the Transplant Trial Watch. The Transplant Trial Watch is a monthly overview of 10 new randomised controlled trials (RCTs) and systematic reviews. This page of Transplant International offers commentaries on methodological issues and clinical implications on two articles of particular interest from the CET Transplant Trial Watch monthly selection. For all high quality evidence in solid organ transplantation, visit the Transplant Library: www.transplantlibrary.com.

RANDOMISED CONTROLLED TRIAL 1 Randomized Trial of a Third Dose of mRNA-1273 Vaccine in Transplant Recipients

*by Hall, V. G., et al. New England Journal of Medicine 2021; 385 (13): 1244–1246.*

## Aims

This study aimed to investigate the effect of a third dose of the mRNA1273 (Moderna) vaccine in solid organ transplant (SOT) recipients.

## Interventions

Participants were randomised to receive either a third dose of mRNA1273 vaccine or saline placebo.

## Participants

120 adult solid organ transplant recipients.

## Outcomes

The primary endpoint was a serologic response. Secondary endpoints included the percent neutralization, and the polyfunctional T-cell response.

## Follow-up

4 months.

## CET Conclusion

Published as a correspondence letter in the NEJM, this study randomised transplant recipients to a third coronavirus vaccine dose (moderna) or to placebo. 120 transplant recipients were included, with median age 66 years and median time after transplant to third vaccine dose of 3 years. Making use of the supplemental appendix and protocol, we can see that this study was conducted in an adequately blinded and randomised fashion, with allocation concealment. Four months after the third injection, 55% in the study group and 18% in the placebo group had antibody levels over the threshold of 100 U/ml for anti-receptor-binding domain antibodies. There was also a significant improvement in virus neutralisation in the study group (71% vs 13%). The third moderna vaccine dose showed a significantly higher immunogenicity than placebo in this patient group, but the authors acknowledge that the study was not powered to, nor had long enough follow up, to assess clinical outcomes.

## Jadad Score

5.

RANDOMISED CONTROLLED TRIAL 2 Conversion from Calcineurin Inhibitor to Belatacept-based Maintenance Immunosuppression in Renal Transplant Recipients: a Randomized Phase 3b Trial

*by Budde, K., et al. Journal of the American Society of Nephrology 2021 [record in progress].*

## Data Analysis

Per protocol analysis.

## Allocation Concealment

Yes.

## Trial Registration

ClinicalTrials.gov–NCT04885907.

## Funding Source

Non-industry funded.

## Aims

Participants were randomised to either the belatacept conversion group or the CNI continuation group.

## Interventions

Participants were randomised into two groups: the intervention group, in which the patients participated in a personalised exercise rehabilitation program in addition to standard care, or the control group where the patients received standard care alone.

## Participants

446 stable adult kidney transplant recipients.

## Outcomes

The primary outcome was the percentage of patients surviving with a functioning graft. Secondary outcome included patient survival, graft survival, incidence and severity of biopsy-proven acute rejection (BPAR), renal function, mean changes in systolic and diastolic blood pressure, proportion of patients with preexisting donor-specific antibodies, and adverse events.

## Follow-Up

24 months.

## CET Conclusion

This large multicentre phase 3b study randomised renal transplant recipients 6–60 months post-transplant to continue CNI, or to switch to Belatacept-based immunosuppression. The primary endpoint (survival with a functioning graft at 24 months) did not differ between groups. There was, however, a clinically significant superior GFR in the Belatacept arm with lower rate of *de novo* DSA, tempered by numerically higher acute rejection rates. It should be noted that the population recruited is relatively low risk, with no recent acute rejection, stable function and EBV seropositive due to risk of PTLD. In reality, the study is underpowered to demonstrate non-inferiority for the primary endpoint, although outcomes in both arms in this respect were excellent. Longer-term follow-up will be interesting to see, as it is quite possible that the improvements in graft function and reduction in dnDSA seen will translate to better long-term graft survival.

## Jadad Score

3.

## Data Analysis

Strict intention-to-treat analysis.

## Allocation Concealment

Yes.

## Trial Registration

ClinicalTrials.gov–NCT01820572.

## Funding Source

Industry funded.

## Clinical Impact Summary

This is a large, well-conducted RCT that took place across multiple centres in several countries. The included patients were adult recipients of both live and deceased donor kidney transplants who were stable 6–60 months after surgery. Patients were also excluded if they had experienced: antibody-mediated rejection at any time; any form of rejection within 3 months of the study start; recurrent acute rejection; greater than or equal to Banff grade-IIA acute rejection in the current allograft; previous graft loss due to BPAR; or had a positive T cell crossmatch prior to the current transplant. Randomisation was adequate and included only one form of stratification; by GFR so that an equal distribution of low functioning kidneys was entered into each study arm. Renal biopsies were mandated in any suspected acute rejection episode, surveillance biopsies were not done.

The primary endpoint was graft survival at 24 months and this was assessed in an intention to treat analysis to preserve randomisation. The withdrawals, dropouts and cross-overs are described and in any event were at tolerable levels for a study of this size. Due to the lack of prior data on graft function on which to base power calculations, the study was powered to exclude significant differences in graft survival instead.

The results show that there was a similar patient and graft survival at 24 months after randomisation and there was no significant difference in acute rejection rates or overall adverse events. However, there was a significant and evolving improvement in eGFR in patients in the belatacept group from baseline, compared to a decline in eGFR in the CNI group. The paper demonstrates a lower proportion of patients that developed *de novo* DSA in the belatacept group than the CNI group, but there is no statistical analysis presented for this outcome.

This study shows that low-risk, stable renal transplant recipients can be converted to belatacept from CNI-based immune suppression with comparable graft survival at 2 years. Whilst there were some post-conversion rejection events, they did not lead to any graft loss in this study and were successfully treated with steroids. Conversion to belatacept in this low-risk population is associated with an improvement renal function and provides a safe option in patients who are intolerant of CNI. If the follow up for the study could be extended, then this improvement in function might also be associated with improved graft survival.

